# Bone Marker Proteins in Women With and Without Polycystic Ovary Syndrome

**DOI:** 10.3390/ijms262110273

**Published:** 2025-10-22

**Authors:** Benjamin M. L. Atkin, Thozhukat Sathyapalan, Laura Dempsey, Stephen L. Atkin, Alexandra E. Butler

**Affiliations:** 1 Edinburgh Royal Infirmary, Old Dalkeith Rd., Edinburgh EH16 4SA, UK; ben.atkin@nhs.scot; 2 Academic Endocrinology, Diabetes and Metabolism, Hull York Medical School, Hull HU6 7RX, UK; thozhukat.sathyapalan@hyms.ac.uk; 3 Royal College of Surgeons in Ireland, D02 YN77 Dublin, Ireland; laura@gmail.com; 4 Royal College of Surgeons in Ireland Bahrain, Busaiteen P.O. Box 15503, Bahrain; satkin@rcsi.com

**Keywords:** polycystic ovary syndrome, bone markers, obesity, periostin, cathepsin

## Abstract

Hormonal alterations associated with polycystic ovary syndrome (PCOS) also impact bone metabolism, though it is unclear if this is bone-protective or not. Bone marker dysfunction has been reported in PCOS and appears to be associated with obesity. This study sought to determine whether a panel of bone marker proteins (BMPs) would be dysregulated in PCOS stratified by BMI as a potential biomarker for bone in PCOS. In this exploratory cross-sectional study, plasma was collected from 234 women (137 with PCOS and 97 controls) from a biobank cohort and compared to a nonobese, non-insulin resistant population (24 with PCOS and 24 controls). Slow Off-rate Modified Aptamer (SOMA)-scan plasma protein measurement was undertaken for the following BMPs: sclerostin; Dickkopf-related protein-1; glycogen synthase kinase-3 alpha/beta; periostin; tumor necrosis factor ligand superfamily member 11; fibroblast growth factor 23; sphingosine kinase 1; sphingosine kinase 2; cathepsins A, B, D, E, G, L2, S and Z; parathyroid hormone; osteocalcin; tumor necrosis factor ligand superfamily member 11 (sRANKL) and interleukin-1 beta. Four BMPs differed in the PCOS cohort (whole set without matching for body mass index (BMI) or insulin resistance (IR)): periostin (*p* = 0.05), cathepsin L (*p* = 0.05) and osteocalcin (*p* = 0.02) decreased in PCOS, whilst cathepsin D (*p* = 0.02) increased; however, linear regression showed that only cathepsins D and L and osteocalcin differed. None of the BMPs differed in the nonobese women with and without PCOS, nor in obese PCOS and controls stratified by BMI greater than 30 kg/m^2^. In subgroup analysis, periostin (*p* = 0.001), sphingosine kinase 2 (*p* = 0.01) and cathepsin L (*p* = 0.001) were higher in obese versus nonobese PCOS (*p* = 0.01). Cathepsin Z (*p* = 0.02), sphingosine kinase 2 (*p* = 0.04) and lysosomal protective protein (*p* = 0.05) were lower in obese versus nonobese controls. Changes in BMPs indicative of impaired bone physiology were associated with BMI in both controls and PCOS, but did not differ between women with and without PCOS when BMI was matched. Hyperandrogenemia in PCOS did not affect BMP levels.

## 1. Introduction

Polycystic ovary syndrome (PCOS) is a common endocrine-metabolic disorder in women of reproductive age with an unclear etiology, characterized by infertility, irregular menstrual cycles, hirsutism, and acne [1]. PCOS is also associated with metabolic features with an increased risk of type 2 diabetes (T2DM), hypertension and early-onset cardiovascular disease (CVD) [2,3]. The underlying pathophysiology of PCOS leading to these conditions is still unclear. Obesity frequently co-occurs with PCOS and is associated with adverse reproductive outcomes [4]. Systemic, low-level inflammation and insulin resistance (IR), both common features of obesity and exacerbated by the PCOS phenotype might be the main mediators of PCOS pathophysiology [5,6]. The prevalence and phenotype of PCOS varies considerably according to ethnicity with, for example, women from the Middle East having a higher prevalence and a more metabolic phenotype [7].

There is debate as to whether PCOS may impact upon bone metabolism, either in a protective or detrimental fashion, with studies on bone mineral density (BMD) suggesting a positive effect [8], a negative effect [9] or no effect [10]. In a large population based study in Denmark, there was a protective effect of PCOS on fracture rates [11] but, conversely, in a Chinese population there was a negative effect of PCOS on fracture rates [12]; however, the two populations differed in terms of hyperandrogenism, body mass index (BMI) and insulin resistance.

Obesity may be related to bone protection with only a decrease in lumbar BMD being seen in normal weight PCOS [13], and lower femur and spine BMD particularly being found in those women with PCOS with a BMI of less than 27 kg/m^2^ [14] and perhaps a BMI of less than 25 kg/m^2^ [15], data suggesting that the increased BMI associated with PCOS may have a protective effect on BMD. Hyperandrogenism may be protective in women with PCOS. In women with hypothalamic amenorrhea associated with low estrogen levels, low BMD was reported but, in women with PCOS, their BMD was not lowered and femoral neck BMD was positively associated with circulating androgens [16].

IR has been positively correlated to BMD [13] and, in a clinical study, women with PCOS had higher BMD compared to amenorrheic women without PCOS, and insulin levels correlated to BMD suggesting that insulin resistance and hyperinsulinemia in women with PCOS may be a relative protective factor against bone mineral loss [17]. Conversely, there is evidence that IR may play a key role in the increased fracture risk observed in both obesity and type 2 diabetes (T2DM), two conditions prevalent in women with PCOS [18].

Chronic inflammation is reported to negatively impact upon BMD [19] with an increased risk of osteoporosis with leptin deficiency and increased oxidative stress [20]. In women with PCOS and inflammation, this has been shown to offset the protective effects of increased weight and muscle mass on bone strength [19].

**Figure 1:**
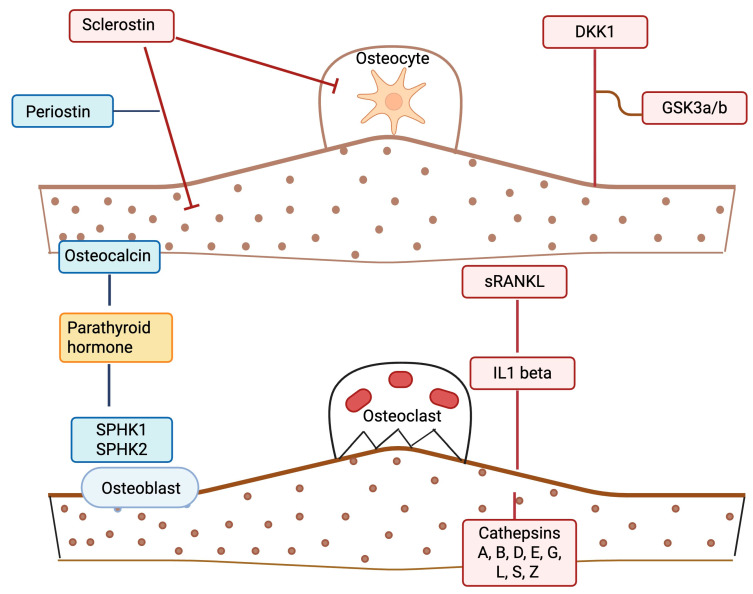
A schematic illustrating potential bone marker protein effects in polycystic ovary syndrome (PCOS). Proteins depicted in blue are anabolic/formation factors. Proteins depicted in red are catabolic/formation factors. The protein in yellow (parathyroid hormone (PTH)) is a mixed regulatory factor. Straight lines indicate a stimulatory effect; blunt-ended lines indicate an inhibitory effect. The illustration was created using BioRender.com (with publication license).

Given the complexity and uncertainty around the effect of PCOS on bone, and the fact that it is difficult to account statistically for BMI with the associated IR and chronic inflammation seen in obesity, this study was undertaken in weight stratified patients with and without PCOS, including a cohort of age- and BMI-matched nonobese women with and without PCOS and without chronic inflammation, to determine if a panel of bone marker proteins differed between the cohorts. Figure 1 details the potential modes of action of the BMPs in the panel.

## 2. Results

Baseline data for the PCOS biobank of 146 PCOS patients and 97 controls are shown in Table 1a and the 24 nonobese, non-insulin-resistant PCOS subjects and controls are shown in Table 1b. For the obese cohort, age was matched, but PCOS subjects had a greater BMI and showed increased insulin resistance, hyperandrogenemia with an elevated testosterone and FAI and increased CRP (a marker of inflammation). For the nonobese, non-insulin-resistant cohort, age and BMI were matched, and the women with PCOS were not insulin-resistant nor was their CRP elevated, but they did have hyperandrogenemia.

The results of the BMP factors are shown in Table 2 for the PCOS biobank cohort and PCOS versus controls (whole set without matching for BMI or IR). Four BMPs differed in PCOS: periostin (*p* = 0.05), cathepsin L2 (*p* = 0.05) and osteocalcin (*p* = 0.02) were decreased in PCOS, whilst cathepsin D was increased in PCOS (*p* = 0.02).

There were no differences in the BMPs in the nonobese age and BMI matched cohort (Supplementary Table S1).

When stratified for BMI greater than 30 kg/m^2^ for both PCOS and controls (demographic data shown in Supplementary Table S2), there were no differences in BMI, CRP, insulin or Homeostasis model of assessment—insulin resistance (HOMA-IR), but the PCOS cohort had hyperandrogenemia with raised testosterone. There were no differences in the BMPs in the BMI > 30 kg/m^2^ PCOS and control cohort (Table 3).

When stratified for PCOS, BMI greater than 30 kg/m^2^ and BMI less than 26 kg/m^2^ (demographic data shown in Supplementary Table S3) there were no differences in CRP, insulin, HOMA-IR, or hyperandrogenemia, and only BMI differed significantly. Periostin, sphingosine kinase 2 and cathepsin L2 were higher in PCOS > 30 kg/m^2^ (*p* = 0.001, *p* = 0.01 and *p* = 0.001, respectively) (Table 4).

When stratified for controls, BMI greater than 30 kg/m^2^ and BMI less than 26 kg/m^2^ (demographic data shown in Supplementary Table S4), there were no differences in CRP, insulin, HOMA-IR, or hyperandrogenemia, and only BMI differed significantly. Cathepsin Z (*p* = 0.02), sphingosine kinase 2 (*p* = 0.04) and lysosomal protective protein (*p* = 0.05) were higher in controls <26 kg/m^2^ (Table 5).

In the adjusted linear regression models, for most biomarkers there was no evidence of a difference between groups. Cathepsin D was higher in the PCOS group (*p* = 0.02), and cathepsin L (*p* = 0.04) and osteocalcin (*p* = 0.05) were lower compared to controls. Periostin (*p* = 0.07) and cathepsin Z (*p* = 0.08) were no longer significant though suggested a trend (Table 6).

## 3. Discussion

Bone marker proteins differed significantly between women with and without PCOS in the PCOS biobank cohort in whole-group analysis with changes in periostin, cathepsin L, osteocalcin and cathepsin D, but linear regression showed changes in cathepsins D and L and osteocalcin, with a trend for periostin and cathepsin Z. Tentatively this could suggest a shift to impaired bone formation and increased resorptive activity that would be in accord with reported studies suggesting that overall in PCOS, there is decreased BMD [9,13]. In this PCOS cohort, there was increased BMI, IR and systemic inflammation with increased CRP and hyperandrogenemia that may have worked in concert to negatively affect bone compared to that of the normal control population. This study is novel, as there is scant data on these BMPs in PCOS-related bone physiology and there is no study measuring all these BMPs at the same time, or synthesizing how all their potential activities may affect bone in PCOS.

Periostin is secreted by osteoblasts, promotes osteoblast differentiation and survival, enhances mechano-sensing in mechanical loading, enhances Wnt/β-catenin signaling, and contributes to collagen crosslinking and matrix organization [21,22]. Therefore, the lower levels seen in whole group analysis may suggest impaired bone formation, and a reduced adaptive response to mechanical stress is associated with low bone mass, fragility and poor bone quality, even when bone mineral density is not markedly reduced, and is seen in conditions of osteoporosis or reduced bone turnover states [23,24]; however, with linear regression, periostin did not significantly differ but rather may have shown a trend. Periostin has been reported to differ in PCOS compared to controls and to be positively correlated with BMI, IR and CRP, and it is suggested that it may be linked to ovarian function [25]; however, others have suggested that its levels do not differ in PCOS [26]. Its levels following weight stratification or linear regression have not previously been determined, nor has its role in PCOS bone physiology been defined.

Cathepsin L is a cysteine protease expressed in osteoclasts and some osteoblast lineage cells that contributes to degradation of bone matrix proteins [27,28], It is decreased in both whole group and linear regression analysis. If it is decreased this may suggest reduced proteolytic activity of osteoclasts, suggesting lower bone resorption capacity that may contribute to low bone turnover states or imbalance in bone remodeling [29]. Cathepsin L is not extensively studied in humans, but is found in human granulosa cells and may be increased by luteinizing hormone and may be involved in follicle rupture [30]. Its role in PCOS bone physiology has not been determined to date.

Osteocalcin is a non-collagenous protein secreted by osteoblasts during bone matrix synthesis and is reported to be a marker of bone formation and osteoblast activity [31]. It also may function as a hormone influencing insulin sensitivity and testosterone production [32]. Lower levels were seen in whole-group analysis and with linear regression. A decrease may reflect reduced osteoblast activity and bone formation [33]. Osteocalcin wasnoted to be markedly reduced in PCOS women with a BMI < 27 kg/m^2^, but did not differ in those with a BMI > 27 kg/m^2^ [34].

Increases in cathepsin D were seen for both whole-group and linear analysis. Cathepsin D is a lysosomal aspartyl protease expressed in multiple tissues, including osteoclasts. In bone, it is reported to degrade non-collagenous proteins and facilitates osteoclastic bone resorption [35]. Its increase may suggest enhanced osteoclastic activity and bone resorption, and elevated cathepsin D activity is associated with collagen breakdown and matrix degradation [35]. Cathepsin D expression has been noted to be down-regulated in ovaries and is found in the endometrium of PCOS patients [30,36,37], but its role in PCOS bone physiology has not been determined to date.

In the age- and nonobese-BMI-matched cohort with and without PCOS, these PCOS women did not have IR or systemic inflammation (as CRP was not elevated), but did have hyperandrogenemia. There were no differences in any of the BMPs, suggesting that there was no effect on BMPs by hyperandrogenemia alone. This would seem incongruous to the reports of a decrease in BMD in normal-weight PCOS [13,14,15]; however, this cohort was unusual in not having IR or systemic inflammation, both of which are associated with a decreased BMD [17,18,19].

When BMI stratification was undertaken for the PCOS (BMI > 30 kg/m^2^) versus controls (BMI > 30 kg/m^2^) there were no differences in BMI, IR or systemic inflammation, though the PCOS cohort remained hyperandrogenemic. There were no changes in BMPs suggesting that the changes in BMPs seen were due to obesity and its associated pathophysiological sequalae of increased IR and systemic inflammation and that hyperandrogenism alone in the presence of obesity did not affect the BMP levels, similarly to the result seen for the nonobese cohort detailed above.

Subgroup analysis with BMI stratification was undertaken for the PCOS (BMI > 30 kg/m^2^) and PCOS (BMI < 26 kg/m^2^) subjects the only differences were in BMI, with IR, systemic inflammation and hyperandrogenemia being no different. Periostin, sphingosine kinase 2 and cathepsin L were decreased in the cohort with a BMI less than 26 kg/m^2^. As detailed above both periostin and cathepsin L decreases could suggest a shift to impaired bone formation and increased resorptive activity. Sphingosine kinase 2 produces sphingosine-1-phosphate (S1P), a bioactive lipid mediator that is critical in cell survival, migration, angiogenesis, and bone remodeling [38], but has not been reported in PCOS patients to date. In bone, S1P regulates osteoblast–osteoclast coupling and bone turnover. If sphingosine kinase 2 is decreased, then hypothetically that may result in reduced S1P production leading to impaired bone cell signaling. In the osteoblast, there is reduced bone formation and mineralization capacity, whilst in the osteoclast there is potential dysregulation of osteoclast apoptosis, leading to prolonged resorptive activity [38]. Overall, this triad may potentially lead to bone remodeling toward bone fragility with low formation and relatively higher resorption, which would be in accord with the decrease in BMD reported in normal weight PCOS [13,14,15]. Conversely, it could be said that periostin, sphingosine kinase 2 and cathepsin L were increased in the cohort with a BMI more than 30 kg/m^2^, which may suggest that increased periostin could be associated with bone turnover and increased bone fragility [23]; an increase in sphingosine kinase 2 promotes osteoclast recruitment and potential bone resorption and is implicated in the osteolytic process [39]; an increase in cathepsin L may indicate active osteoclast-mediated bone resorption [40], thus the triad may hypothetically be detrimental to bone homeostasis. Thus, with IR, systemic inflammation and hyperandrogenemia being accounted for between cohorts, BMI appears to be the major factor leading to the change in the BMPs.

Subgroup analysis with BMI stratification was undertaken for the control (BMI > 30 kg/m^2^) and control (BMI < 26 kg/m^2^) subjects; the only difference was in BMI, with IR, systemic inflammation and hyperandrogenemia being no different. Sphingosine kinase 2, cathepsin Z and lysosomal protective protein (cathepsin A) were lower in those with a BMI greater than 30 kg/m^2^. Cathepsin Z is a cysteine protease with a unique RGD integrin-binding domain that may facilitate osteoclast adhesion, migration, matrix degradation and osteoblast precursor adhesion. Reduction results in osteoclasts that attach less effectively to bone surface, leading to impaired resorption [41]. However, in linear analysis of the PCOS biobank cohort, sphingosine kinase 2, cathepsin Z and lysosomal protective protein (cathepsin A) were not found to differ significantly.

PCOS is a complex condition that has inherent independent effects on clinical parameters as well as indirect effects, such as with concomitant obesity, resulting in multicollinearity that may distort linear analysis. In addition, regression analysis assumes a binary outcome, rather than the continuous data that is the case for each BMP, and regression analysis assumes that the data is linear, which is often not the case with real world data; this may have resulted in differences between the analyses. We believe that presenting both approaches, subgroup comparisons for clinical clarity and regression analyses for statistical robustness, offers the most complete picture of the data. Therefore, the changes in periostin cathepsin L, osteocalcin and cathepsin D are preliminary findings and need be treated with some caution until functional validation or direct correlation data with bone mass can be undertaken. Bone mineral density was not undertaken in either of the cohorts; therefore, it was not possible to correlate the BMPs with this important parameter. Additional limitations of this study include that it was performed on a Caucasian population and would need to be repeated to consider ethnic differences. PCOS phenotype A, which expresses all three of the diagnostic criteria (as for the PCOS biobank cohort), is reported to be at higher risk of adverse metabolic and cardiovascular outcomes compared to the other phenotypes, and phenotype D is the least severe. All the nonobese PCOS subjects had anovulatory infertility, but half were phenotype B (irregular menses with hyperandrogenism) and half were phenotype C (irregular menses and polycystic ovaries on transvaginal scanning).There were too few subjects to compare the groups. Thus, expression of BMP-related proteins needed to be clarified for the individual PCOS phenotypes. A strength of this study was the BMI stratification of obese and nonobese PCOS and controls, to account for BMI, IR, systemic inflammation and hyperandrogenemia, as adjusting for BMI and insulin resistance statistically is very difficult as both are so highly correlated with PCOS that regression adjustment for either or both may remove the PCOS effects. Future larger studies with this BMP panel, together with measures of BMD and bone strength, are needed to determine their utility as biomarkers for bone physiology in weight-stratified PCOS patients, and whether any of these BMPs may be of utility as bone fragility markers in PCOS.

In conclusion, in the biobank PCOS and control cohorts, BMPs showed an indicative shift to impaired bone formation and increased resorptive activity in PCOS. In the weight-matched and -stratified obese and nonobese PCOS, hyperandrogenemia alone had no effect on BMPs. When obese and nonobese PCOS were compared, changes in BMPs were indicative of bone remodeling, potentially toward bone fragility. When obese and nonobese controls were compared, indicative changes in BMPs towards suppressed bone remodeling in the obese control group were toward bone fragility, potentially with both formation and resorption being impaired.

## 4. Materials and Methods

### 4.1. Study Cohorts

#### 4.1.1. Obese PCOS and Control Group [42]

Plasma levels of BMPs were measured in women diagnosed with polycystic ovary syndrome (PCOS; *n* = 137) and healthy controls (*n* = 97), all of whom were recruited from a UK-based PCOS Genetic Biobank (ISRCTN70196169). Written informed consent was obtained from all participants. The study was approved by the Newcastle & North Tyneside Research Ethics Committee.

All participants were of Caucasian ethnicity. PCOS was diagnosed based on at least two of the three Rotterdam criteria, as previously detailed [43]: (1) clinical and/or biochemical hyperandrogenism (Ferriman–Gallwey score > 8, free androgen index > 4, or total testosterone > 1.5 nmol/L), (2) oligo- or amenorrhea, and (3) polycystic ovarian morphology on transvaginal ultrasound. Alternative diagnoses including nonclassical 21-hydroxylase deficiency, hyperprolactinemia, Cushing’s syndrome, and androgen-secreting tumors were excluded through appropriate investigations. Baseline clinical measurements have been previously described [44]. Control participants, recruited via public advertisement, reported regular menstrual cycles (28 ± 4 days), showed no clinical or biochemical signs of hyperandrogenism, had normal ovarian morphology (i.e., had no features of PCOS), had no significant comorbidities and were not taking any prescription or over-the-counter medications, including hormonal contraceptives.

#### 4.1.2. Nonobese PCOS and Control Group [45]

BMPs were also measured in a separate cohort of nonobese women with PCOS (*n* = 24) and BMI- and age-matched controls (*n* = 24) recruited from the Hull IVF clinic [46]. Inclusion criteria included age between 20 and 40 years and a body mass index (BMI) ≤ 30 kg/m^2^. Control women were undergoing IVF for male factor infertility or unexplained infertility. All eligible participants attending the clinic for a routine mock embryo transfer procedure were approached, and those who agreed provided written informed consent. This study received ethical approval from the Yorkshire and the Humber National Research Ethics Service (NRES), UK (approval number: 02/03/043).

#### 4.1.3. Sample Processing and Biochemical Analysis

Fasting blood samples were centrifuged at 3500× *g* for 15 min, aliquoted, and stored at –80 °C until analysis. Measurements included C-reactive protein (CRP), sex hormone-binding globulin (SHBG), insulin (DPC Immulite 200 analyzer, Euro/DPC, Llanberis, UK), and plasma glucose (Synchron LX20 analyzer, Beckman-Coulter, High Wycombe, UK). Free androgen index (FAI) was calculated as: FAI = (Total Testosterone/SHBG) × 100. Insulin resistance was assessed using the homeostasis model assessment (HOMA-IR). Serum testosterone concentrations were measured using isotope-dilution liquid chromatography tandem mass spectrometry (LC-MS/MS)(TSQ Altis Plus Triple Quadrupole Mass Spectrometer, Thermofisher, Altrincham, UK) [46].

Circulating BMP levels were quantified using the SOMAscan proteomic platform (Version 3.1, Somalogic, Boulder, CO, USA), based on Slow Off-rate Modified Aptamer (SOMA) technology [47]. The SOMAscan assay version 3.1 was used, with normalization, hybridization, and calibration performed as per manufacturer’s specifications using internal standards on each plate. Details of the assay have been previously reported [48].

Targeted BMPs [41,49,50,51] included sclerostin, Dickkopf-1, glycogen synthase kinase-3α/β, periostin, tumor necrosis factor ligand superfamily member 11 (sRANKL), fibroblast growth factor 23, sphingosine kinases 1 and 2, multiple cathepsins (A, B, D, E, G, L2, S, Z), parathyroid hormone, osteocalcin, and interleukin-1β (refer to Figure 1 and Table 2 for detailed listings).

### 4.2. Statistical Analysis

A formal power analysis is the preferred approach in study design; however, the absence of prior data on expected effect sizes meant a reliable a priori calculation was not feasible. Therefore, we conducted this study as an exploratory pilot, with the primary aim of generating initial estimates of variability and effect sizes to guide future studies. Data distributions were visually and statistically assessed for normality using the Kolmogorov–Smirnov statistical test, because for all proteins the *p* value of the K-S test was greater than 0.05, indicating the likely normal distribution of the data; Student’s *t*-test was used to compare differences between groups. Parametric analyses (independent *t*-tests) were applied to normally distributed variables, while non-parametric comparisons (Mann–Whitney U tests) were used for variables that did not meet normality assumptions. A *p* value of 0.05 or less was taken to be statistically significant. For statistical analysis, Graphpad Prism v9.5.1 (San Diego, CA, USA) was utilized.

Individual linear regression models were used to examine differences in BMP levels between women with PCOS and controls in the biobank cohort. Each model included group (PCOS vs. control) and was adjusted for age and BMI. Least squares mean (LS-mean) differences (PCOS–control) with 95% confidence intervals (CIs) were estimated for each BMP. Additional models including group x BMI interaction were also tested, but none of the interactions were statistically significant, indicating that there was no evidence that the association between PCOS status and BMPs differed by BMI. These analyses were completed using SAS version 9.4 (SAS Institute Inc., Cary, NC, USA).

## Figures and Tables

**Table 1:** (**a**) Demographics and baseline hormonal and metabolic parameters of the polycystic ovary syndrome biobank (PCOS) subjects and controls. (**b**) Demographics and baseline hormonal and metabolic parameters of the age- and BMI-matched PCOS subjects and controls (mean ± SD). All parameters did not differ, other than those marked ** = *p* < 0.01.

(**a**)		
**Baseline Demographics**	**PCOS (*n* = 137)**	**Controls (*n* = 97)**
	**Mean (SD)**	**Mean (SD)**
Age (Years)	29.1 ± 6.1	29.6 ± 6.5
BMI (Kg/m^2^)	34.1 ± 7.5	26.7 ± 6.6 ***
Insulin (IU/mL)	10.2 ± 6.1	6.2 ± 3.2 ***
HOMA-IR	3.8 ± 0.6	1.6 ± 0.2 ***
Testosterone (nmol/L)	1.6 ± 1.0	1.05 ± 0.48 ***
SHBG (nmol/L)	42.5 ± 39.6	77.5 ± 78.4 ***
Free androgen index (FAI)	4.5 ± 3.9	2.1 ± 1.4 ***
CRP (mg/L)	4.4 ± 4.2	2.4 ± 3.9 ***
AMH (ng/mL)	40 ± 31	18 ± 18 ***
(**b**)		
	**PCOS (*n* = 24)**	**Control (*n* = 24)**
Age (years)	31.0 ± 6.4	32.5 ± 4.1
BMI (kg/m^2^)	25.9 ± 1.8	24.8 ± 1.1
Insulin (IU/mL)	8.1 ± 4.7	7.7 ± 4.0
HOMA-IR	1.9 ± 1.6	1.8 ± 1.0
Testosterone (nmol/L)	1.4 ± 0.8	0.7 ± 0.4 ***
SHBG (nmol/L)	71.7 ± 62.2	104 ± 80
Free androgen index (FAI)	4.1 ± 2.9	1.3 ± 0.5 **
CRP (mg L^−1^)	2.8 ± 2.6	2.3 ± 2.3
AMH (ng/mL)	57.0 ± 14.0	24.0 ± 13.0 **

BMI—body mass index; HOMA-IR—homeostasis model of assessment—insulin resistance; CRP— C-reactive protein; SHBG—sex hormone binding globulin; AMH—anti-Müllerian hormone. ** *p* < 0.01, *** *p* < 0.001.

**Table 2:** Bone marker proteins for the polycystic ovary syndrome (PCOS) biobank cohort. PCOS *n* = 137, controls *n* = 97, without body mass index (BMI) stratification. Data presented as Mean ± 1 Standard Deviation of Relative Fluorescent Units (RFU).

	Control	PCOS	*p* Value
	Mean	Mean	
Sclerostin	17,492 ± 6445	16,633 ± 7141	0.34
Dickkopf-related protein 1	21,810 ± 10,375	22,557 ± 11,375	0.60
Glycogen synthase kinase-3 alpha/beta	6307 ± 5672	5773 ± 4095	0.39
Periostin	5171 ± 1128	4853 ± 1292	0.05
Tumor necrosis factor ligand superfamily member 11 (sRANKL)	1419 ± 9359	519 ± 431	0.25
Fibroblast growth factor 23	578 ± 378	579 ± 357	0.98
Sphingosine kinase 1	3336 ± 3734	2918 ± 2416	0.29
Sphingosine kinase 2	498 ± 432	468 ± 266	0.51
Cathepsin Z	4377 ± 1071	4600 ± 1058	0.11
Cathepsin G	869 ± 384	972 ± 551	0.11
Cathepsin B	1260 ± 382	1224 ± 257	0.39
Cathepsin S	903 ± 208	937 ± 224	0.30
Cathepsin L	1548 ± 498	1418 ± 22	0.05
Cathepsin E	1337 ± 2268	1190 ± 693	0.46
Cathepsin D	626 ± 434	837 ± 781	0.02
Cathepsin H	454 ± 148	510 ± 266	0.06
Lysosomal protective protein	6077 ± 2845	6037 ± 2392	0.91
Parathyroid hormone	2196 ± 76	2344 ± 878	0.18
Osteocalcin	3247 ± 9243	1438 ± 963	0.02
Interleukin-1 beta	3281 ± 2288	3185 ± 2207	0.74

**Table 3:** Weight-stratified polycystic ovary syndrome (PCOS) (Body mass index (BMI) > 30 kg/m^2^) versus controls (BMI > 30 kg/m^2^) for bone marker proteins. Data presented as Mean ± 1 Standard Deviation of Relative Fluorescent Units (RFU).

Protein	PCOS (*n* = 91)	Control (*n* = 19)	
	**Mean**	**Mean**	***p* Value**
Sclerostin	15,394 ± 7298	10,488 ± 8197	0.74
Dickkopf-related protein 1	22,104 ± 11,749	19,114 ± 14,626	0.37
Glycogen synthase kinase-3 alpha/beta	5898 ± 4309	6123 ± 5343	0.98
Periostin	4382 ± 1485	2880 ± 1860	0.26
Tumor necrosis factor ligand superfamily member 11 (sRANKL)	479 ± 452	340 ± 185	0.59
Fibroblast growth factor 23	572 ± 414	405 ± 267	0.62
Sphingosine kinase 1	3017 ± 2591	2996 ± 2403	0.81
Sphingosine kinase 2	415 ± 117	323 ± 208	0.21
Cathepsin Z	4488 ± 1355	3501 ± 2236	0.40
Cathepsin G	993 ± 643	854 ± 678	0.08
Cathepsin B	1172 ± 347	875 ± 5534	0.32
Cathepsin S	898 ± 300	721 ± 446	0.46
Cathepsin L	1281 ± 532	747±491	0.24
Cathepsin E	1149 ± 751	826 ± 462	0.72
Cathepsin D	786 ± 691	679 ± 412	0.24
Cathepsin H	489 ± 319	343 ± 217	0.35
Lysosomal protective protein	5922 ± 2551	4996 ± 3175	0.09
Parathyroid hormone	2200 ± 959	2069 ± 1569	0.77
Osteocalcin	1504 ± 1189	1204 ± 1015	0.60
Interleukin-1 beta	3214 ± 2823	2646 ± 1607	0.52

**Table 4:** Weight stratified polycystic ovary syndrome (PCOS) (Body mass index (BMI) > 30 kg/m^2^) versus PCOS (BMI < 26 kg/m^2^) for bone marker proteins. Data presented as Mean ± 1 Standard Deviation of Relative Fluorescent Units (RFU).

Protein	BMI > 30 kg/m^2^ *n* = 91	BMI < 26 kg/m^2^ *n* = 19	
	**Mean**	**Mean**	***p* Value**
Sclerostin	15,259 ± 7609	7271 ± 6276	0.96
Dickkopf-related protein 1	21,767 ± 12,202	11,290 ± 9267	0.33
Glycogen synthase kinase-3 alpha/beta	5918 ± 4383	3516 ± 2685	0.85
Periostin	4201 ± 1619	1849 ± 1178	0.001
Tumor necrosis factor ligand superfamily member 11 (sRANKL)	474 ± 464	278 ± 222	0.34
Fibroblast growth factor 23	570 ± 425	303 ± 240	0.39
Sphingosine kinase 1	3029 ± 2591	1854 ± 1386	0.85
Sphingosine kinase 2	401 ± 129	175 ± 174	0.01
Cathepsin Z	4313 ± 1486	1921 ± 1875	0.14
Cathepsin G	950 ± 545	523 ± 414	0.88
Cathepsin B	1131 ± 376	498 ± 490	0.25
Cathepsin S	860 ± 321	393 ± 374	0.35
Cathepsin L	1186 ± 517	545 ± 504	0.001
Cathepsin E	1147 ± 774	586 ± 479	0.54
Cathepsin D	787 ± 706	481 ± 373	0.50
Cathepsin H	476 ± 329	250 ± 202	0.82
Lysosomal protective protein	5781 ± 2666	2807 ± 2457	0.31
Parathyroid hormone	2194 ± 992	1104 ± 952	0.37
Osteocalcin	1444 ± 1101	842 ± 641	0.52
Interleukin-1 beta	3195 ± 2886	1861 ± 1439	0.41

**Table 5:** Weight-stratified controls (Body mass index (BMI) < 26 kg/m^2^) versus obese controls (BMI > 30 kg/m^2^) for bone marker proteins. Data presented as Mean ± 1 Standard Deviation of Relative Fluorescent Units (RFU).

Protein	BMI > 30 kg/m^2^ (*n* = 19)	BMI < 26 kg/m^2^ (*n* = 59)	
	**Mean**	**Mean**	***p* Value**
Sclerostin	6483 ± 5609	1249 ± 7484	0.91
Dickkopf-related protein 1	1017 ± 8532	21,230 ± 13,762	0.33
Glycogen synthase kinase-3 alpha/beta	3179 ± 2552	5334 ± 3806	0.58
Periostin	1671 ± 1552	3583 ± 1900	0.33
Tumor necrosis factor ligand superfamily member 11 (sRANKL)	241 ± 202	418 ± 411	0.56
Fibroblast growth factor 23	266 ± 221	474 ± 318	0.85
Sphingosine kinase 1	1655 ± 1310	2679 ± 1936	0.67
Sphingosine kinase 2	162 ± 153	332 ± 159	0.04
Cathepsin Z	1765 ± 1648	3715 ± 1885	0.02
Cathepsin G	467 ± 383	843 ± 519	0.11
Cathepsin B	453 ± 427	919 ± 448	0.34
Cathepsin S	359 ± 330	736 ± 367	0.06
Cathepsin L	483 ± 441	949 ± 528	0.29
Cathepsin E	515 ± 436	921 ± 498	0.68
Cathepsin D	410 ± 330	772 ± 843	0.43
Cathepsin H	219 ± 183	429 ± 414	0.93
Lysosomal protective protein	2529 ± 2221	5256 ± 2893	0.05
Parathyroid hormone	1003 ± 871	1988 ± 1147	0.09
Osteocalcin	745 ± 596	1087 ± 661	0.26
Interleukin-1 beta	1636 ± 1334	2626 ± 1285	0.42

**Table 6:** Bone marker proteins in women with and without polycystic ovary syndrome (PCOS)—additional regression analysis. Individual linear regression models were used to examine differences in bone marker protein (BMP) levels between women with PCOS and controls in the biobank cohort. Each model included group (PCOS vs. control) and was adjusted for age and body mass index (BMI). Least squares mean (LS-mean) differences (PCOS—control) with 95% confidence intervals (CIs) were estimated for each BMP.

	Difference in LS-Means (PCOS—Control) [95% CI]	*p*-Value
Sclerostin	−400.39 [−2065.98, 1265.20]	0.64
Dickkopf-related protein 1	224.24 [−2820.80, 3269.28]	0.88
Glycogen synthase kinase-3 alpha/beta	224.24 [−2820.80, 3269.28]	0.88
Periostin	−312.29 [−654.16, 29.58]	0.07
Tumor necrosis factor ligand superfamily member 11	−937.89 [−2631.59, 755.81]	0.28
Fibroblast growth factor 23	16.22 [−73.61, 106.06]	0.72
Sphingosine kinase 1	−385.19 [−1228.76, 458.38]	0.37
Sphingosine kinase 2	−36.67 [−132.04, 58.70]	0.45
Cathepsin Z	248.06 [−42.63, 538.75]	0.09
Cathepsin G	105.77 [−32.82, 244.37]	0.13
Cathepsin B	−23.04 [−109.47, 63.39]	0.60
Cathepsin S	25.97 [−32.82, 84.76]	0.38
Cathepsin L	−137.91 [−272.51, −3.32]	0.04
Cathepsin E	−150.94 [−586.94, 285.07]	0.50
Cathepsin D	210.46 [36.23, 384.69]	0.02
Cathepsin H	61.22 [−2.95, 125.39]	0.06
Cathepsin A	−55.92 [−771.52, 659.67]	0.88
Parathyroid hormone	192.06 [−41.14, 425.25]	0.11
Osteocalcin	−1692.95 [−3373.63, −12.26]	0.05
Tumor necrosis factor ligand superfamily member 11	−937.89 [−2631.59, 755.81]	0.28
Interleukin-1 beta	−92.86 [−727.43, 541.71]	0.77

## Data Availability

All the data for this study will be made available upon reasonable request to the corresponding author.

## Supplementary Materials

The following supporting information can be downloaded at: https://www.mdpi.com/article/10.3390/ijms262110273/s1.
