# Supplementary material for: Bone Marker Proteins in Women With and Without Polycystic Ovary Syndrome
Source: Int J Mol Sci. 2025 Oct 22;26(21):10273. doi: 10.3390/ijms262110273 (PMC12607462; doi:10.3390/ijms262110273)
Supplement: Supplementary file 1 [file ijms-26-10273-s001.zip › ijms-3872288-Supplementary tables.pdf]

Supplementary Table S1. Bone marker proteins for the PCOS matched for age, BMI, systemic resistance cohort. PCOS (n=24), controls (n=24).
Data presented as Mean ± 1 Standard Deviation of Relative Fluorescent Units (RFU).

	PCOS		Control		
	Mean	SD	Mean	SD	p value
Sclerostin	12057.2	5991.6	11228.4	3983.9	0.54
Dickkopf-related protein 1	25251.9	15613.9	24325.7	13496.7	0.81
Glycogen synthase kinase-3 alpha/beta	6341.8	8460.5	4598.8	2562.0	0.29
Periostin	3786.1	1076.9	3789.0	1155.1	0.99
Tumor necrosis factor ligand superfamily member 11 (sRANKL)	404.7	183.1	368.8	158.0	0.43
Fibroblast growth factor 23	427.7	109.0	510.3	253.3	0.11
Sphingosine kinase 1	3865.2	5981.9	2232.9	1172.1	0.16
Sphingosine kinase 2	352.0	59.9	347.3	102.2	0.83
Cathepsin Z	4424.3	1130.3	4310.9	1023.6	0.69
Cathepsin G	956.6	396.0	1172.9	993.3	0.28
Cathepsin B	1230.7	244.4	1216.9	386.4	0.87
Cathepsin S	814.8	166.1	1007.8	727.0	0.17
Cathepsin L	1899.6	661.1	1826.1	759.2	0.70
Cathepsin E	381.3	106.5	447.6	284.1	0.24
Cathepsin D	636.8	339.3	2518.3	7738.4	0.20
Cathepsin H	760.1	216.6	798.2	397.8	0.65
Lysosomal protective protein	5843.3	2820.5	6031.0	2801.9	0.80
Parathyroid hormone	2030.1	739.4	1849.3	895.6	0.41
Osteocalcin	1688.0	3587.2	1434.1	919.3	0.71
Interleukin-1 beta	2315.8	784.6	2491.8	1057.0	0.48

Supplemental Table S2. Demographics, baseline, hormonal and metabolic parameters of the PCOS subjects (BMI>30 kg/m^2, n=91) and controls (BMI>30 kg/m^2, n=19) (mean±SD). All parameters did not differ other than those marked **=p<0.01.

BMI >30 kg/m^2 PCOS vs Controls	PCOS >30 kg/m^2		Controls >30 kg/m^2		p value
	Mean	SD	Mean	SD	
Age (years)	28.5	6.1	29.8	6.0	0.39
BMI (kg/m^2)	37.4	5.9	36.8	6.8	0.69
CRP (mg/L)	4.7	4.3	3.8	6.1	0.49
Baseline glucose (mmol/l)	4.9	1.2	4.7	0.4	0.36
Insulin (IU/ml)	10.4	6.4	7.1	5.7	0.35
HOMA-IR	3.7	6.8	1.3	0.8	0.20
Testosterone (nmol/L)	1.7	1.1	1.0	0.3	0.009**

Supplemental Table S3. Demographics, baseline, hormonal and metabolic parameters of the PCOS subjects (BMI>30 kg/m^2, n=91) and PCOS subjects (BMI<26 kg/m^2, n=19) (mean±SD). All parameters did not differ other than those marked **=p<0.01.

PCOS BMI<26 kg/m^2 vs BMI >30 kg/m^2	PCOS>30 kg/m^2		PCOS<26 kg/m^2		
	Mean	SD	Mean	SD	p value
Age (years)	28.5	6.1	29.8	6.5	0.35
BMI (kg/m^2)	37.4	5.9	23.1	2.1	0.000001**
CRP (mg/L)	4.7	4.3	2.2	3.4	0.90
Baseline glucose (mmol/l)	4.9	1.2	4.7	0.8	0.29
Insulin (IU/ml)	10.4	6.4	6.8	2.9	0.69
HOMA-IR	3.7	6.8	1.9	1.9	0.85
Testosterone (nmol/L)	1.7	1.1	1.1	0.5	0.18

Supplemental Table S4. Demographics, baseline, hormonal and metabolic parameters of the control subjects (BMI>30 kg/m^2, n=19) and control subjects (BMI<26 kg/m^2, n=59) (mean±SD). All parameters did not differ other than those marked **=p<0.01.

Control BMI<26 kg/m^2 vs BMI >30 kg/m^2	Control >30 kg/m^2		Control <26 kg/m^2		
	Mean	SD	Mean	SD	p value
Age	29.8	6.0	29.8	6.5	0.99
BMI (kg/m^2)	36.8	6.8	23.1	2.1	0.000001**
CRP mg/L	3.8	6.1	2.2	3.4	0.19
Baseline glucose (mmol/l)	4.7	0.4	4.7	0.8	0.90
Insulin (IU/ml)	7.1	5.7	6.8	2.9	0.86
HOMA-IR	1.3	0.8	1.9	1.9	0.29
Testosterone (nmol/L)	1.0	0.3	1.1	0.5	0.37
